# STRATEGIES FOR ENGAGING LOW-INCOME SENIOR HOUSING RESIDENTS IN INTERDISCIPLINARY WELLNESS CLINICS

**DOI:** 10.1093/geroni/igad104.0599

**Published:** 2023-12-21

**Authors:** Sarah Holmes, Jennifer Klinedinst, Nicole Brandt, Barbara Resnick

**Affiliations:** University of Maryland School of Nursing, Baltimore, Maryland, United States; University of Maryland School of Nursing, Baltimore, Maryland, United States; University of Maryland, Baltimore, Baltimore, Maryland, United States; University of Maryland, Baltimore, Maryland, United States

## Abstract

Engaging residents in low-income senior housing communities in health and wellness services can be challenging. Despite the significant healthcare needs of residents in senior housing communities, numerous barriers to engaging residents exist including the mistrust of academic or healthcare institutions, stigma about health conditions and treatment, and lack of awareness about services available. To successfully implement Interdisciplinary Wellness Clinics, a participatory approach was used through engagement of stakeholders in the housing communities. The Interdisciplinary Wellness Clinic team worked closely with the Senior Program Manager and the community and regional property managers to establish a plan for implementation. The needs of the communities, day and timing of the clinics that would fit with their schedules, and a plan for ways to invite and engage the residents was developed. Flyers were made by the interdisciplinary team for visits at each community and sign-up sheets for the services provided on any given month. The flyers were posted on bulletin boards within the communities or in a central location for residents to access them by the community property managers. Intermittently, associated with holidays for example, we also held raffles for gifts for anyone who attended the clinic. Regular meetings were held with the property managers at each of the senior housing communities to solicit their input and brainstorm strategies for continued engagement of residents and identify new approaches to increase engagement in wellness clinic services.

